# Polyclonal Antibody Therapeutics: Analytical Innovations and Regulatory Perspectives for Addressing Heterogeneity Challenges

**DOI:** 10.1021/acs.analchem.5c06531

**Published:** 2026-02-19

**Authors:** Sunil Kumar, Aurora Tini, Sara Tengattini, Francesca Rinaldi, Enrica Calleri, Gabriella Massolini, Caterina Temporini

**Affiliations:** Department of Drug Sciences, University of Pavia, Viale Taramelli 12, 27100 Pavia, Italy

## Abstract

Polyclonal antibodies (pAbs) are a cornerstone of the adaptive immune system, providing broad-spectrum protection by recognizing and neutralizing diverse antigens. Intravenous immunoglobulins (IVIg) derived from animal/human plasma are widely used to treat diseases such as primary immunodeficiencies, autoimmune neurological disorders, and situations requiring rapid immune protection, such as emerging infectious disease outbreaks. Compared with monoclonal antibodies (mAbs), pAbs offer several advantages, including faster, less technically demanding, and more cost-effective production, often directly from immunized animals or human serum. However, their complex, heterogeneous nature—reflecting the polyclonal response to multiple epitopes—creates significant challenges for characterization, quality control, and regulatory approval. pAbs have been underutilized in therapeutic applications due to the absence of robust, standardized analytical tools capable of fully assessing their heterogeneity. Conventional chromatographic and spectroscopic methods yield limited qualitative and quantitative information, insufficient for detailed structural and functional evaluation. This perspective highlights recent advances in high-resolution analytical technologies, particularly chromatographic and mass spectrometric approaches, that enable in-depth characterization of critical quality attributes (CQAs) of pAbs. These analytical strategies facilitate the assessment of parameters such as structural integrity, molecular size distribution, subclass composition, and polyclonality, which are crucial for ensuring product quality. Alongside these technical developments, regulatory frameworks for pAb-based therapeutics are evolving, emphasizing the need for standardized analytical criteria to ensure safety, efficacy, and batch-to-batch consistency. In our view, by integrating new analytical capabilities with clear regulatory expectations, pAbs can be more effectively developed as therapeutic agents, complementing mAbs and expanding the range of immunoglobulin-based interventions in clinical practice.

## Graphical abstract

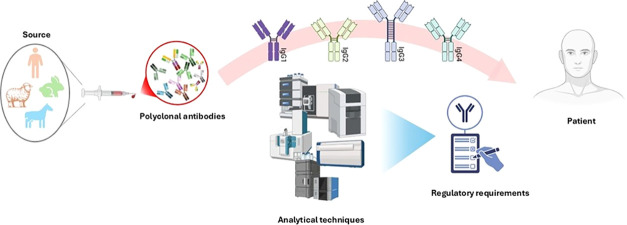

## 1 Introduction

Immunoglobulins, or antibodies, are present in plasma and extracellular fluids, where they act as key effectors of the adaptive immune system. They provide an early defense against foreign agents such as bacteria, viruses, malignant cells.^1^ Antibody-derived biotherapeutics generally fall into two major categories: monoclonal antibodies (mAbs) and polyclonal antibodies (pAbs). In addition to their therapeutic applications in cancer, autoimmune disorders, and bacterial or viral infections,^2^ antibodies are widely used as diagnostic agents (e.g., in tests for hepatitis, HIV, and COVID-19 antigens) and as essential research tools in assays such as ELISA, Western blotting, and immunohistochemistry. mAbs are produced by a single B-lymphocyte clone (commonly via hybridoma or recombinant expression systems) and therefore consist of a single antibody type and epitope specific, even though they have some degree of heterogenicity linked to post translational modifications. By contrast, pAbs are a more heterogeneous population of antibodies because they are secreted by multiple B-lymphocyte clones in response to a single antigenic stimulus and then collected from plasma. Consequently, pAbs recognize and bind to multiple, distinct epitopes on the same antigen, providing broad reactivity and enhanced overall avidity.^3^

Immunoglobulins (Igs) consist of two identical light chains (two types namely “κ” and “λ”), including two domains each, and two identical heavy chains, usually with four or five domains each. Mammalian B-cell can generate five antibodies’ isotypes—IgM, IgG, IgA, IgD, and IgE (differentiated by the types of heavy chain; γ, μ, α, δ, and ε)—each tailored to distinct immunological functions.^4,5^ More structural details on Igs are given in the next paragraph. During a primary immune response, immature B cells predominantly secrete IgM; upon re-exposure to antigen, they undergo isotype (class) switching to IgG, which typically exhibits higher affinity. Additional class switching occurs in mucosal tissues, where IgA predominates to provide barrier protection from pathogens due to immune exclusion.^6^ Meanwhile, IgE primarily mediates immunity against parasites, but it also plays a key role in allergic disorders (type I hypersensitivity).^7^ IgD, although present only at low concentrations in the circulation, plays a regulatory role in B-cell activation and signaling.^8^ Furthermore, in certain mammalian species—including humans, mice, and rabbits—IgG and IgA antibodies are divided into distinct subclasses (e.g., IgG1–IgG4 and IgA1–IgA2 in humans).

Antibodies, mainly IgG, are pivotal in humoral immunity due to their abundance and multifaceted effector functions, including neutralization of pathogens, activation of the classical complement pathway, and engagement of Fc receptors to mediate phagocytosis, antibody-dependent cellular cytotoxicity (ADCC) and Immunothrombosis. Fc receptor expressed mainly on platelets can trigger a mechanism immunothrombosis which leads to platelets activation and aggregation, release of procoagulant and inflammatory mediator, expression of phosphatidylserine that promotes thrombin generation and activation of the inflammatory response.^9,10^ Additionally, the neonatal Fc receptor (FcRn) plays a crucial role in extending the serum half-life of IgG through pH-dependent recycling, which is particularly beneficial in therapeutic contexts. IgG antibodies can be utilized as polyclonal preparations, offering a broad spectrum of antigen recognition, or as mAbs, providing high specificity for targeted therapies. Both forms are integral to the development of vaccines and biologic drugs, with ongoing advancements in engineering to enhance efficacy and safety profiles.

Plasma-derived pAbs remain the preferred therapeutic option for the management of selected acute medical emergencies because they provide broad and physiologically relevant immune protection. Despite their longstanding clinical use, a comprehensive overview of analytical approaches to characterize quality attributes of pAb products are currently lacking. This perspective article seeks to fill this gap by offering an introductory overview of current advances in plasma-derived IgG products analysis. Specifically, it focuses on the structural features of IgG and its subclasses, highlights current analytical strategies for IgG characterization, and discusses ongoing challenges in pAbs quality control. In addition, the article reviews relevant regulatory expectations and offers a forward-looking assessment of emerging analytical trends shaping the future of plasma-derived antibody therapeutics.

## 2 Characteristics of Immunoglobulins (IgGs)

*Structure of IgG*: IgG, the predominant serum antibodies (≈10–20% of plasma proteins), are subdivided into four subclasses (IgG1–IgG4) ranked by decreasing abundance and distinct functional profiles.^11^ Structurally, all IgG subclasses, consist of four polypeptide chains—two identical light chains (∼25 kDa) and two identical heavy chains (∼50 kDa)—linked together by interchain disulfide bonds. Each heavy chain consists of three primary components: an N-terminal variable domain (VH) and three constant domains (CH1, CH2, CH3), and an extra “hinge region” between CH1 and CH2. Likewise, the light chain consists of N-terminal variable domain (VL) and a constant domain (CL). Each light chain associates with the variable (VH) and first constant (CH1) domains of a heavy chain via a disulfide bond, forming the fragment antigen-binding (Fab) arm responsible for antigen recognition. The remaining portion of the antibody, comprising the lower hinge region together with the CH2 and CH3 domains of the heavy chains, constitutes the fragment crystallizable region (Fc), which mediates effector functions such as complement activation and interaction with Fc receptors.^12^ On the heavy chains, IgG bears a highly conserved N-linked glycosylation site at asparagine 297 (N297) within the CH2 domain of the Fc region (Figure 1). In addition, approximately 10–20% of Fab regions also contain N-glycosylation sites introduced through somatic hypermutation. The predominant glycans attached to these sites are complex-type structures rich in mannose and N-acetylglucosamine (GlcNAc) residues.^13^ These N-glycans can be variably galactosylated, sialylated, or fucosylated, generating distinct glycoforms. Such modifications critically influence antibody’s biological activity, particularly its anti-inflammatory properties; for example, Fc N-glycan variants show a graded increase in anti-inflammatory activity from agalactosylated (G0) to digalactosylated disialylated (G2S2) forms.^14^ In addition to N-glycans, O-glycans can also be found in hinge region attached to Threonine/Serine of IgG3.^15,16^ The four IgG subclasses share more than 90% amino-acid sequence identity overall, they differ markedly in the hinge region and the N-terminal portion of the CH2 domain, while the remaining domains display fewer differences. All these structural variations confer distinct biological properties to each subclass, including differences in serum half-life, antigen-binding characteristics, placental transfer, complement activation, immune-complex formation, and cell-triggering capacity. Moreover, different antigens tend to elicit preferential responses from specific IgG subclasses. Although selective subclass deficiencies are generally asymptomatic, they can increase susceptibility to certain pathogens in some individuals.^11^

**Figure 1:**
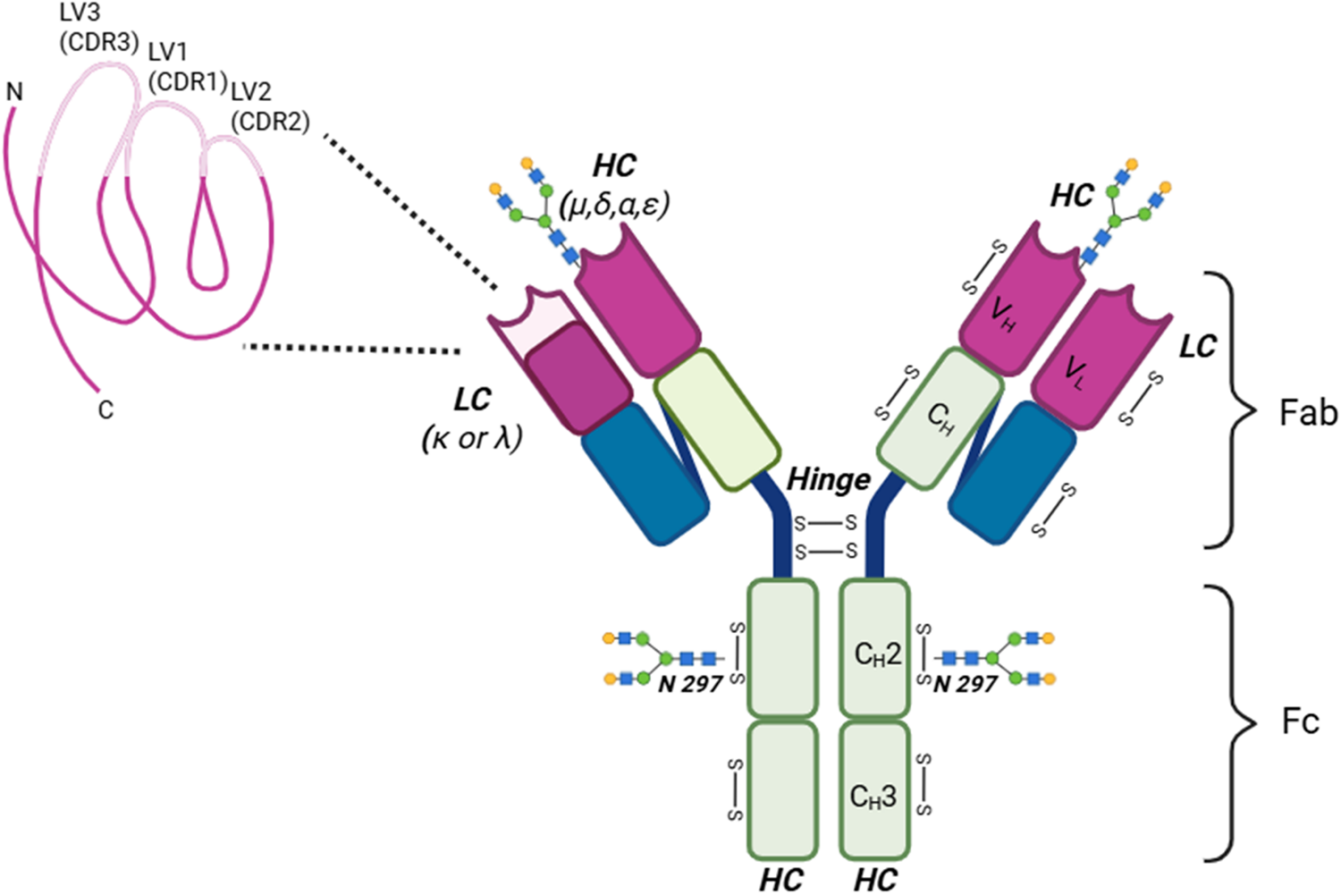
Schematic illustration of IgG depicting its characteristic Y-shaped configuration formed by heavy and light chains.

### 2.1 Subclasses of IgG and Nomenclature

The type of antigen and the associated immune context critically influence the generation of the four IgG subclasses.^17,18^ Despite their overall structural similarity, the IgG subclasses differ markedly in hinge composition and interchain disulfide architecture, differences that substantially affect their flexibility and effector functions^11^ (see Table 1). IgG1 is distinguished by the disulfide bond that links heavy and light chains: the carboxy-terminal cysteine of the light chain is covalently connected to the cysteine at position 220 in the CH1 domain of the heavy chain, whereas in IgG2, IgG3, and IgG4 the linkage occurs at cysteine 131.^19^ This canonical HC–LC linkage contributes to the typical stability and geometry of IgG1 molecules. Among subclasses, IgG2 exhibits greater structural heterogeneity owing to hinge isomers that result from alternative disulfide bond arrangements between cysteines in the hinge and those that connect the heavy and light chains.^19^ These isomers are observed more often in the presence of κ light chains than λ light chains. The two predominant IgG2 forms are the A form, with four inter–heavy-chain disulfide bonds, and the B form, in which one hinge cysteine instead links to a light chain.^20^ Despite these differences in disulfide topology, FcRn binding appears to be preserved across IgG2 isomers.^21^ IgG2 can also form covalent dimers, representing an additional isomeric variant.^22^ Among the subclasses, IgG3 has the longest and most disulfide-rich hinge, with up to 11 inter–heavy-chain disulfide linkages.^11^ Hinge length also varies among IgG3 allotypes, reflecting greater evolutionary divergence. The exceptional hinge length and its disulfide architecture markedly influence the spatial arrangement and mobility of the Fab arms and Fc tail. These conformational properties can partially or completely shield complement component C1q or Fcγ-receptor binding sites on the Fc region, thereby modulating complement activation and Fcγ-receptor engagement.^23^ In the case of IgG4, it is notable for its propensity to adopt alternative disulfide arrangements in the hinge. Its core CPSC motif favors intrachain disulfide formation between cysteines at positions 226 and 229,^24^ allowing IgG4 to exist both as the conventional covalently linked H_2_L_2_ molecule and as noncovalently associated “half-molecules” (HL). These unique structural behaviors underlie IgG4′s distinct functional profile and its association with immune tolerance and responses to chronic antigenic stimulation. IgG4 differs from other IgG subclasses by its labile heavy chain–heavy chain interactions, allowing dissociation into half-molecules and subsequent in vivo and in vitro half-molecule exchange, generating novel bivalent antibodies.^25^ Together, these subclass-specific differences in hinge length and disulfide connectivity shape IgG structural dynamics and directly influence effector functions, including complement activation, Fc-receptor interactions, and downstream immune outcomes. The structural distinctions among the subclasses have been summarized in Table 1.

**Table 1:**
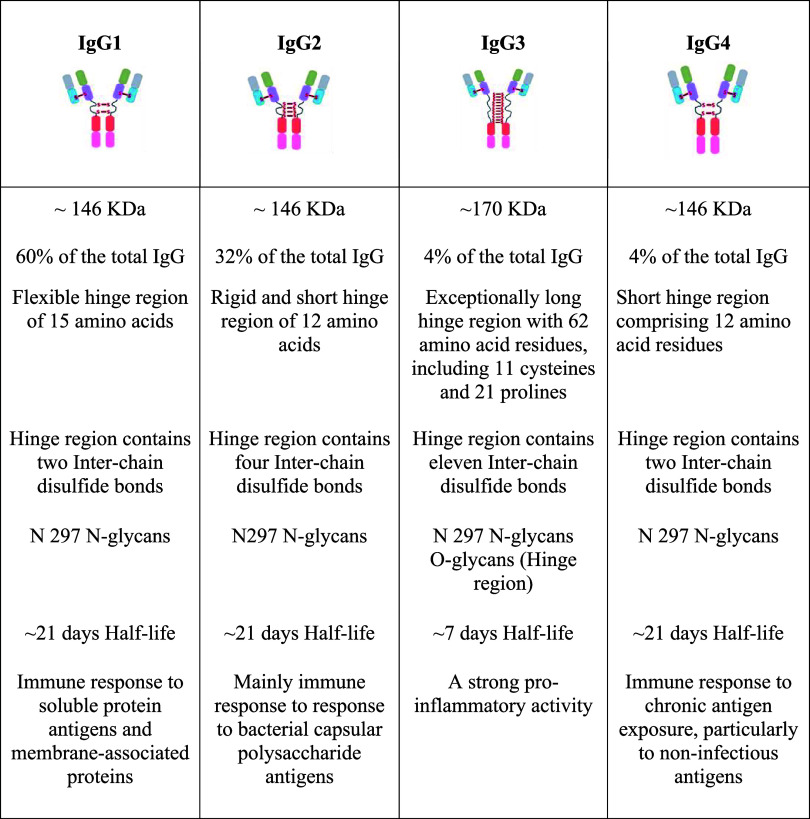
Structural Features of Human IgG Subclasses

### 2.2 Allotypes of IgG

Beyond subclass-specific structural differences, human IgG molecules also exhibit allotypic variation, defined by inherited antigenic differences detectable serologically. These allotypes occur on both the κ light chains and the heavy chains of IgG1, IgG2, and IgG3. Their distribution and frequency vary across populations, reflecting underlying genetic diversity. To date, four heavy-chain allotypes have been characterized for IgG1, one for IgG2, and as many as 13 for IgG3.^26^ Such polymorphism extends well beyond serological markers, influencing Fc receptor binding, effector functions, and immunogenicity.^27–29^ Consequently, careful selection of IgG allotypes is a key consideration in the design and development of therapeutic antibodies intended for human use.

Summarizing, subclasses IgG1, IgG2, IgG3, and IgG4 can be differentiated on the basis of the size of the hinge region, position of interchain disulfide bonds, molecular weight and variation in the heavy chain like sequence variation.

### 2.3 Polyclonal and Monoclonal Antibodies: Key Differences

Although mAbs currently dominate the landscape of therapeutic interventions, pAbs continue to play a critical role in both research and clinical applications. Unlike mAbs, which are typically generated against specific peptides, pAbs are generally produced against native proteins or protein fragments. This difference in antigen targets necessitates a more stringent validation process for pAbs to ensure consistency and reliability in experimental outcomes.^30,31^ From a biophysical perspective, the inherent heterogeneity of pAbs can confer enhanced stability under environmental stressors. Nevertheless, heterogeneity may arise during the production and purification processes, where differences in handling, fractionation, or recovery can further contribute to the diversity of antibody species within a lot. Consequently, lot-to-lot variability can occur due to differences in the relative composition of antibody subclasses, which reflect both the host immune response during immunization and the combined effects of environmental and physiological factors during antibody production. Such variability can impact on the overall binding properties, effector functions, and biophysical behavior of the final pAb preparation. Therefore, by systematically characterizing multiple lots, manufacturers can identify and control sources of variability, leading to more reliable and consistent pAbs products. Table 2 summarizes the principal differences between mAbs and pAbs in terms of production methods, epitope recognition profiles, and therapeutic applications.

**Table 2:**
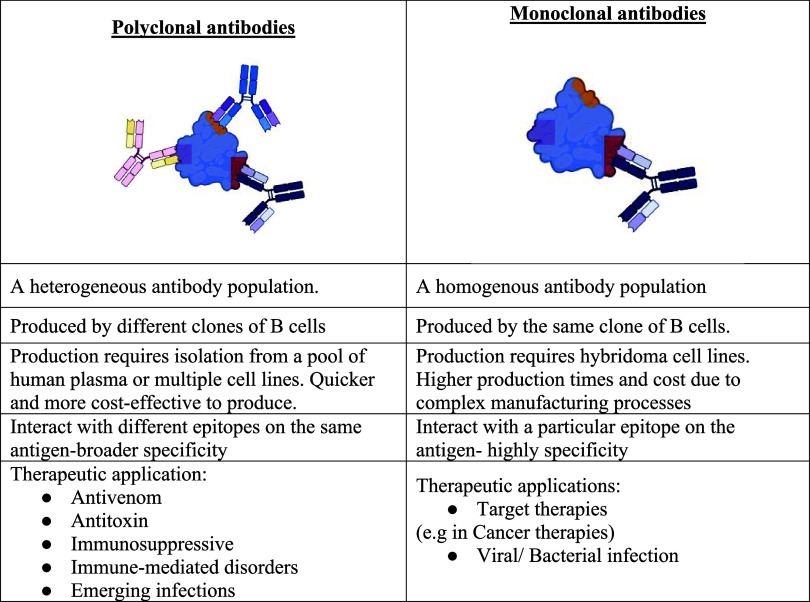
Comparative Overview of Polyclonal Antibodies (pAbs) and Monoclonal Antibodies (mAbs), Highlighting Key Differences in Their Production Methods, Epitope Recognition, and Therapeutic Applications

### 2.4 Production and Therapeutics Application of Immunoglobulins (Polyclonal IgG)

Plasma-derived immunoglobulins from animals and humans have a well-established history of clinical use for therapeutic purposes.^30^ In advantages: they can be generated at high titers, produce broad immune responses, and provide a scalable and cost-effective source of plasma for therapy. However, the administration of these heterologous products frequently resulted in adverse reactions, due to immunogenic incompatibility.^32,33^ Contemporary immunoglobulin therapeutics are predominantly derived from pooled human plasma, collected from either healthy donor—typically from 10,000 to 15,000 donors—or individuals with elevated antibody titers following natural infection or immunization against specific pathogens. These plasma-derived products contain targeted antibodies directed against specific antigens (95% IgG), in addition to a substantial proportion of polyclonal, nonspecific immunoglobulins.^34^ The utilization of human IgG depends on donor availability as well as potential safety issues linked to plasma-derived products.^35^

Therapeutic pAbs, such as intravenous immunoglobulin (IVIg), undergo extensive large-scale industrial purification prior to formulation. IVIg is manufactured from large plasma pools (>1000 L) that are initially frozen to enable cryoprecipitation, followed by an initial ethanol precipitation step. The product subsequently undergoes multiple downstream purification processes, including additional ethanol fractionation, ion-exchange (IEX) chromatography, solvent–detergent treatment, and caprylic acid (CA) precipitation, in conjunction with dedicated virus inactivation and removal procedures. Overall, IVIg manufacturing typically involves 5–10 purification steps in addition to viral safety and formulation stages, resulting in a highly purified drug substance or drug product.^36^

A significant concern with pAbs isolated from animal or human plasma is the considerable lot-to-lot variability observed during production. This variability can arise from the collection of blood serum at different times from the same host or from different hosts altogether, leading to differences in the composition of pAbs lots. For antibody manufacturers, this poses a challenge, as producing consistent lots of pAbs requires ongoing production and validation of new antibody batches, along with the development of suitable replacement products. This challenge can be mitigated by the consistent collection of high-quality data that is reproducible across various experiments and research laboratories. To maintain reliability and accuracy, researchers may need to validate protocols or optimize experimental conditions with each new batch of antibodies, especially if certain products become unavailable or obsolete. Currently, over 20 FDA-approved pAbs products are available on the market, used in therapies such as antiviral treatments, antitoxins, antivenins, and others.^37^ These FDA-approved pAb products are plasma-derived antibodies obtained from the plasma or serum of immunized hosts, including humans, horses, sheep, and rabbits, following vaccination against the target antigen.

Currently, pAb based therapies are increasingly employed in the management of infectious diseases, owing to their intrinsic polyclonality, which closely mimics the natural immune response. This polyclonality allows simultaneous recognition of multiple epitopes, enhancing viral and toxin neutralization—an advantage demonstrated in established therapies such as snake antivenom, rabies immunoglobulin, and tetanus antitoxin. In addition to their use in infectious diseases, pAbs are widely applied in autoimmune and inflammatory diseases, in immunosuppressive regimens to prevent and treat graft rejection in organ transplantation (e.g., renal and skin allografts) and in the management of immune-mediated disorders such as aplastic anemia and heparin-induced thrombocytophenia.^38–40^

## 3 Regulatory Guideline and Risk Assessments

According to EMA regulatory guidelines, pAb products must undergo thorough quality evaluation, including assessment of molecular size distribution with quantification of monomers, dimers, fragments, polymers, and aggregates; physicochemical, immunochemical properties and analysis of impurities such as other plasma proteins and non-IgG immunoglobulins (e.g., IgA, IgM, IgE)^41^ and one of the most critical aspect-determination of IgG that is subclass distribution.^42^ These guidelines ensure that only consistent, effective, and high-quality products, developed under a highly regulated framework, reach commercialization. Achieving these standards requires a comprehensive analytical toolbox that encompasses a wide range of techniques targeting the diverse physicochemical properties of the product. pAb therapeutics, while clinically valuable, are inherently constrained by their dependence on plasma pooled from numerous donors, which elevates the risk of contamination. A single compromised donation has the potential to contaminate multiple product batches, thus impacting a broad patient population. To mitigate these risks, regulatory authorities require rigorous risk assessment and comprehensive characterization of plasma-derived products, including pAbs used as active substances, investigational agents, excipients, or ancillary components in medical devices. Critical safety evaluations include screening for clinically relevant bacterial pathogens (e.g., *Corynebacterium diphtheriae*, *Haemophilus influenzae* type b, *Streptococcus pneumoniae*, *Streptococcus pyogenes*) and viral pathogens (e.g., hepatitis A and B viruses, cytomegalovirus, varicella-zoster virus, rubella, measles, parvovirus B19, poliovirus type 1). IVIg as part of the parental products needs to be evaluated on endotoxins and pyrogens levels as described in FDA and EMA guidelines.^43–45^ Additional risk mitigation involves assessing anticomplementary activity, determining titers of anti-A and anti-B hemeagglutinins/hemolysins, monitoring anti-D antibody levels, and quantifying prekallikrein activator. Together, these measures ensure product safety, minimize patient risk, and establish robust safeguards against contamination.^46,47^

Beyond viral safety, process validation plays a pivotal role in pAbs characterization, ensuring reproducibility, robustness, and consistency during large-scale manufacturing. Quality control extends to monitoring critical process parameters such as pH, ethanol concentration, and temperature during fractionation, as well as measuring residual solvent and detergent levels in the final formulation. Additional considerations include the controlled use of processing aids (e.g., anticoagulants and adsorbents), accompanied by thorough documentation of residual concentrations to confirm product purity. Stability testing, in compliance with ICH Q5C guidelines, is also required for intermediates and final pAb products to verify integrity and therapeutic efficacy throughout the shelf life.^48^ Collectively, these requirements provide a rigorous regulatory and scientific framework for the characterization of therapeutic polyclonal antibodies, ensuring product consistency, patient safety, and reliable clinical performance. According to regulatory guidelines, analytical approaches to characterize pAb are discussed with details in the next section.

## 4 Analytical Approaches for the Comprehensive Characterization of pAbs

As we have discussed in the previous paragraph, pAb products represent a highly complex class of biological therapeutics, encompassing multiple immunoglobulin isotypes, subclasses, and proteoforms derived from heterogeneous biological sources. This intrinsic diversity necessitates comprehensive analytical characterization to ensure product quality, consistency, and regulatory compliance. Recent advances in analytical technologies have substantially improved the monitoring of critical quality attributes (CQAs) in pAbs. In accordance with ICH Q11, drug substance CQAs are defined as those physical, chemical, biological, or microbiological properties that have a direct impact on product identity, purity, biological activity, and stability, and therefore on safety and efficacy.^49^ The identified attributes may pose a higher potential risk to product safety and efficacy and may therefore be classified as CQAs following a product-specific, risk-based assessment. For instance, product-related impurities such as fragments, aggregates, and process-related contaminants are considered high-risk attributes due to their potential impact on safety, stability, and clinical performance, and therefore require stringent monitoring and control. The polyclonal composition of pAb products represents a key functional quality attribute, as antibody diversity underpins biological activity and therapeutic efficacy. Additionally, antibody subclass distribution is an important quality attribute, given that different IgG subclasses exhibit distinct pharmacokinetic and stability profiles (e.g., IgG1 generally demonstrates a longer half-life than other subclasses), which may influence product shelf life and in vivo performance.

A schematic diagram depicting the various product quality attributes is provided in Figure 2, and the analytical approaches employed to monitor these attributes are described in the following sections. IVIg can be directly analyzed using the described analytical techniques without the need for further purification. The essential sample preparation—such as buffer exchange, dilution, or desalting to ensure compatibility with the analytical platform—may be required, depending on the specific method employed (e.g., LC–MS or native MS). The application of orthogonal, state-of-the-art analytical methods generates a data set that supports comparability assessments, stability monitoring, and lifecycle management of pAb products in alignment with current regulatory expectations.

**Figure 2:**
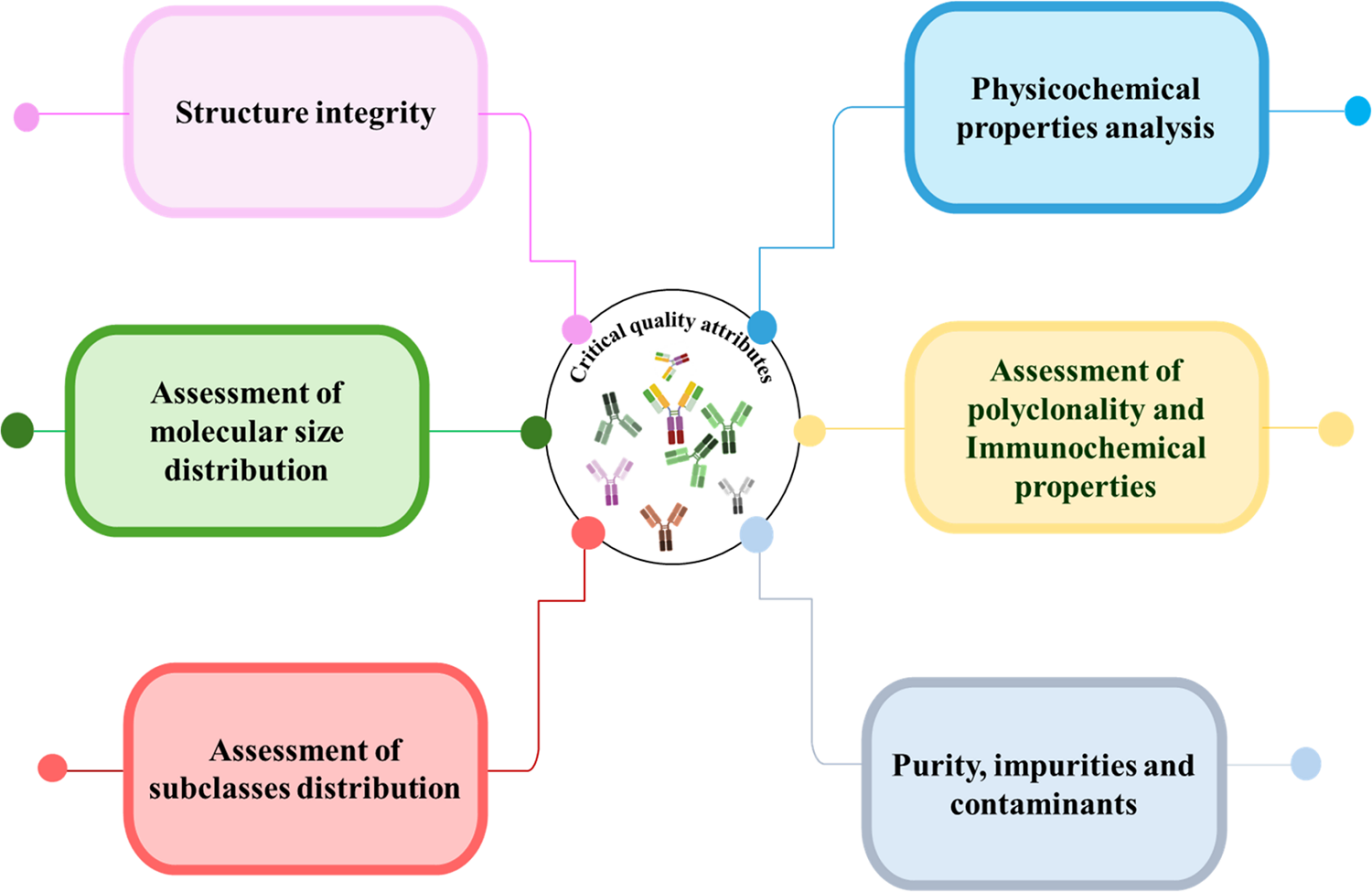
Schematic diagram depicting the various product quality attributes.

### 4.1 Structure Integrity Analysis

The structural integrity of pAbs refers to the preservation of their proper higher-order structures (HOS), including secondary (α-helices, β-sheets, and loops), tertiary (stable polypeptide folding) and quaternary (assembly of heavy and light chains) structures, and their biologically active conformations within the heterogeneous antibody mixture. Structure integrity studies of mAbs are performed with different analytical techniques like circular dichroism (CD), Fourier-transform infrared spectroscopy (FTIR), differential scanning calorimetry (DSC), fluorescence spectroscopy and nuclear magnetic resonance (NMR) spectroscopy, often employed in combination to obtain additional insights into the structural characteristics of antibody products.^50^ While these techniques are widely accepted and routinely applied for the structural characterization of mAbs, to the best of our knowledge, no studies have specifically reported on their application for assessing the structural integrity of pAbs. In the case of pAbs, bulk biophysical methods typically yield ensemble-averaged data across the heterogeneous antibody population and may therefore lack the resolution required to identify subtle conformational differences among individual variants within the polyclonal pool. A strategy integrating multiple orthogonal techniques should be established to capture the complexity of pAbs heterogeneity while compensating for the inherent limitations of each individual assay.

### 4.2 Assessment of Molecular Size Distribution

Molecular size distribution (MSD) is a CQA of therapeutic pAbs, as it defines the relative proportions of aggregates, intact monomers and fragments present in the product. Accurate assessment of MSD is essential for ensuring product quality. Size-exclusion chromatography (SEC) and Capillary Electrophoresis–Sodium Dodecyl Sulfate (CE-SDS) are the most widely employed techniques for this purpose. SEC, often coupled with multiangle light scattering (SEC-MALS), enables separation and quantification of antibody species based on molecular weight under near-physiological conditions, making it particularly effective for detecting monomers, fragments, and oligomers/aggregates. MSD of human IgG preparations has been systematically evaluated in a single study, using four different SEC columns, each varying in particle and pore size, length, and internal diameter.^51^ This study, demonstrated that the selection of modern columns, with smaller particle sizes and thus higher resolution capacities, is essential to resolve the full spectrum of size variants (dimers, trimers, and oligomers), thereby ensuring characterization of all species in human pAb products.^51^ Complementary to SEC, CE-SDS offers high-resolution size-based separation under both reducing and nonreducing conditions, thereby providing insights into product purity and heterogeneity at the molecular level. Frandsen and colleagues developed a recombinant pAb product, rozrolimupab, comprising-Rhesus D (Rh D) specific antibodies produced through a single-batch manufacturing concept that target blood group antigen in substitution to the plasma derived product anti-Rh D immunoglobulin. Using CE, they demonstrated that the product predominantly contained full-length antibody molecules, with only a minor fraction of aggregates detected under nonreducing conditions.^35^ In addition to chromatographic and electrophoretic approaches, recent advances in mass spectrometry (MS), particularly native MS and ion mobility mass spectrometry (IM-MS), have emerged as powerful tools for the analysis of intact antibody species under near-physiological conditions. Native MS has been well established method for characterization of mAbs.^52^ These techniques preserve noncovalent interactions, allowing antibodies to be examined in their native conformations. Consequently, native MS and IM-MS provide detailed insights into molecular heterogeneity, conformational stability, and MSD, offering a deeper understanding of antibody structure (shape, size, and charge state distribution) and higher-order organization.^53,54^ Zheng et al. applied a direct infusion native MS approach to analyze four groups of crude pAb samples isolated from mice, revealing that the monomeric pAb populations exhibited substantial heterogeneity in their molecular distribution.^55^ In addition to analyzing the heterogeneity of pAbs, IM-MS has also been employed to evaluate their conformational and structural stability. A comparative study, using two ion unfolding strategies—the conventional collision-induced unfolding (CIU) and the advanced all-ion unfolding (AIU), has been carried out to quantitatively assess the structural stability of four pAb samples. The AIU approach exhibited markedly superior resolving capability, providing a 2–4-fold enhancement in both stability and structural differentiation parameters relative to the traditional CIU method. This improvement enabled a more accurate and detailed characterization of conformational heterogeneity within pAbs populations.^56^ Furthermore, beyond direct infusion methods, MS can also be coupled to liquid chromatography (LC) systems using different techniques such as SEC, ion-exchange chromatography (IEX), and reverse-phase liquid chromatography (RP-LC), for enhancing separation and resolution of antibody variants prior to the mass spectrometer. Unlike mAbs, pAbs comprise a complex mixture of immunoglobulins with diverse amino acid sequences, leading to highly heterogeneous and often overlapping mass spectra that may present significant challenges for resolution and interpretation. However, when possible, Limited Charge Reduction (LCR) can be used to untangle overlapping charge-state distributions from different protein populations. LCR involves isolating a narrow mass/charge window and inducing controlled charge reduction reactions to stepwise remove protons and create a unique charge reduction ladder for the ions within this window. The distinct ladder patterns make it possible to identify and measure the masses of the contributing species.^57,58^ Furthermore, recent advancements in mass spectrometry instrumentation and data analysis are expected to enhance the sensitivity, resolution, and throughput of methods like all ion unfolding-ion mobility (AIU-IM), which might allow for more precise and comprehensive characterization of pAbs structural heterogeneity.

### 4.3 Assessment of Subclasses Distribution

The Fc region of antibodies harbors most of the structural information that distinguishes isotypes and allotypes, primarily within the CH2 and CH3 domains. To evaluate subclass distribution, middle-up or subunit analysis strategies are commonly employed. In these approaches, antibody heterogeneity is simplified by generating defined fragments through enzymatic digestion or chemical reduction, thereby reducing analytical complexity compared to intact-level analysis. The reduction of interchain disulfide bonds with dithiothreitol (DTT) produces separate heavy- and light-chain fragments, facilitating a more detailed assessment of antibody subclass composition.^59^ One of the earliest applications of the subunit analysis approach was undertaken to identify and quantify the individual antibodies within a recombinant pAb product comprising a 25-antibody mixture, achieved through the separation of light and heavy chains. SEC was employed to separate and collect the light- and heavy-chain fractions. The isolated light-chain fractions were subsequently treated with the endoproteinase Asp-N to generate unique peptides specific to each antibody, enabling their unambiguous identification. Thereafter, light chains were analyzed by RP-LC-MS to determine their molecular masses, and the relative abundance of each antibody was quantified based on the signal intensity of the corresponding light-chain species.^35^

An alternative middle-up analytical approach involves targeted enzymatic digestion at or near the hinge region to generate large, well-defined antibody subunits that are analyzed without further fragmentation. Unlike middle-down approaches, which involve additional gas-phase or solution-phase fragmentation for sequence-level analysis, the middle-up strategy focuses on intact subunit mass measurements. This simplification of antibody structure facilitates the assessment of subclass and isotype distribution. Indeed, the hinge region of IgGs is a flexible segment connecting the antigen-binding Fab domains to the Fc region, particularly susceptible to proteolytic cleavage, resulting in the generation of defined IgG fragments. For instance, papain, a cysteine protease, cleaves above the hinge region; pepsin cleaves below the hinge region within the heavy chains; and the bacterial protease IdeS (immunoglobulin-degrading enzyme of *Streptococcus pyogenes*) displays high specificity for the lower hinge region, rapidly generating well-defined F­(ab′)_2_ and Fc fragments.^60^ Surprisingly, although IdeS does not cleave murine IgG3 and IgG4 subclasses, the bacterial protease SpeB, which also derives from *Streptococcus pyogenes* is able to cleave murine polyclonal IgGs into Fc/2 subunits.^59^ Fragments generated through enzymatic cleavage of the hinge region have been utilized to distinguish among different IgG subclasses and allotypes. Blöchl et al. employed the protease SpeB to selectively cleave murine polyclonal IgGs, generating Fc/2 fragments, which are subclass-specific, that were analyzed using ion-pair RP-LC coupled with high-resolution MS. This approach enabled both quantitative assessment of IgG subclasses and detailed characterization of Fc N-glycosylation variants. The method was validated in serum samples from BALB/c mice immunized with the grass pollen allergen Phl p6, where it revealed immunization-induced shifts in IgG subclass distribution as well as alterations in glycosylation profiles, underscoring its utility for probing immune responses at the molecular level.^59^ In addition to subclass assessment, a comprehensive middle-up strategy has been applied to characterize the molecular heterogeneity of human IgGs, focusing on subclass, allotype, and glycosylation variations. In this study, Sénard et al. isolated human IgGs from the plasma of 5 donors using Fc-specific magnetic beads and subsequently digested them with the enzyme IdeS to generate Fc/2 subunits. These Fc/2 fragments were then analyzed using two orthogonal LC-MS-based approaches: hydrophilic interaction liquid chromatography (HILIC-MS) and CE-MS. HILIC-MS enabled high-resolution profiling of glycoforms, including oxidized variants, while CE-MS provided separation of IgG subclasses and allotypes based on differences in charge and size. By integrating these complementary analyses, the authors demonstrated heterozygosity across five donors, identifying a total of 12 distinct allotypes together with subclass-specific glycosylation signatures.^61^ Although these earlier studies demonstrated the feasibility of middle-up approaches for IgG characterization, they were limited by insufficient separation resolution, sensitivity, and detection limits, which restricted reliable analysis of lower-abundance IgG3 and IgG4 allotypes. To address these challenges, Blöchl et al. developed a nanoscale RP-LC-MS workflow enabling comprehensive subclass- and allotype-specific characterization of Fc proteoforms. In this strategy, polyclonal IgG samples were purified from individual donors and subjected to IdeS digestion to release single-chain Fc subunits (Fc/2). Direct nanoscale RP-LC-MS analysis of these Fc/2 fragments provided the resolution necessary to distinguish nearly isobaric allotypes and subclasses, thereby allowing accurate identification and quantification of proteoforms across all four IgG subclasses.^62^

Figure 3 provides an overview of the different middle-up/subunit strategies utilized to identify and characterize the subclasses of pAbs. Although middle-up proteomics offers substantial advantages in terms of sequence coverage, analytical sensitivity, and accuracy compared to traditional intact mass analysis approaches, it still sacrifices certain benefits. In particular, middle-up methods require more extensive sample processing and do not preserve native chain pairing information, which can be critical for understanding antibody structure and function. Despite these limitations, ongoing middle-up strategies show considerable promise for antibody subclass/isotype discovery, as they enable detailed characterization of variable regions, post-translational modifications (PTMs), and Fc proteoforms. These capabilities support the identification of functionally relevant antibody variants, accelerating the development of therapeutic candidates and improving our understanding of polyclonal antibody heterogeneity.

**Figure 3:**
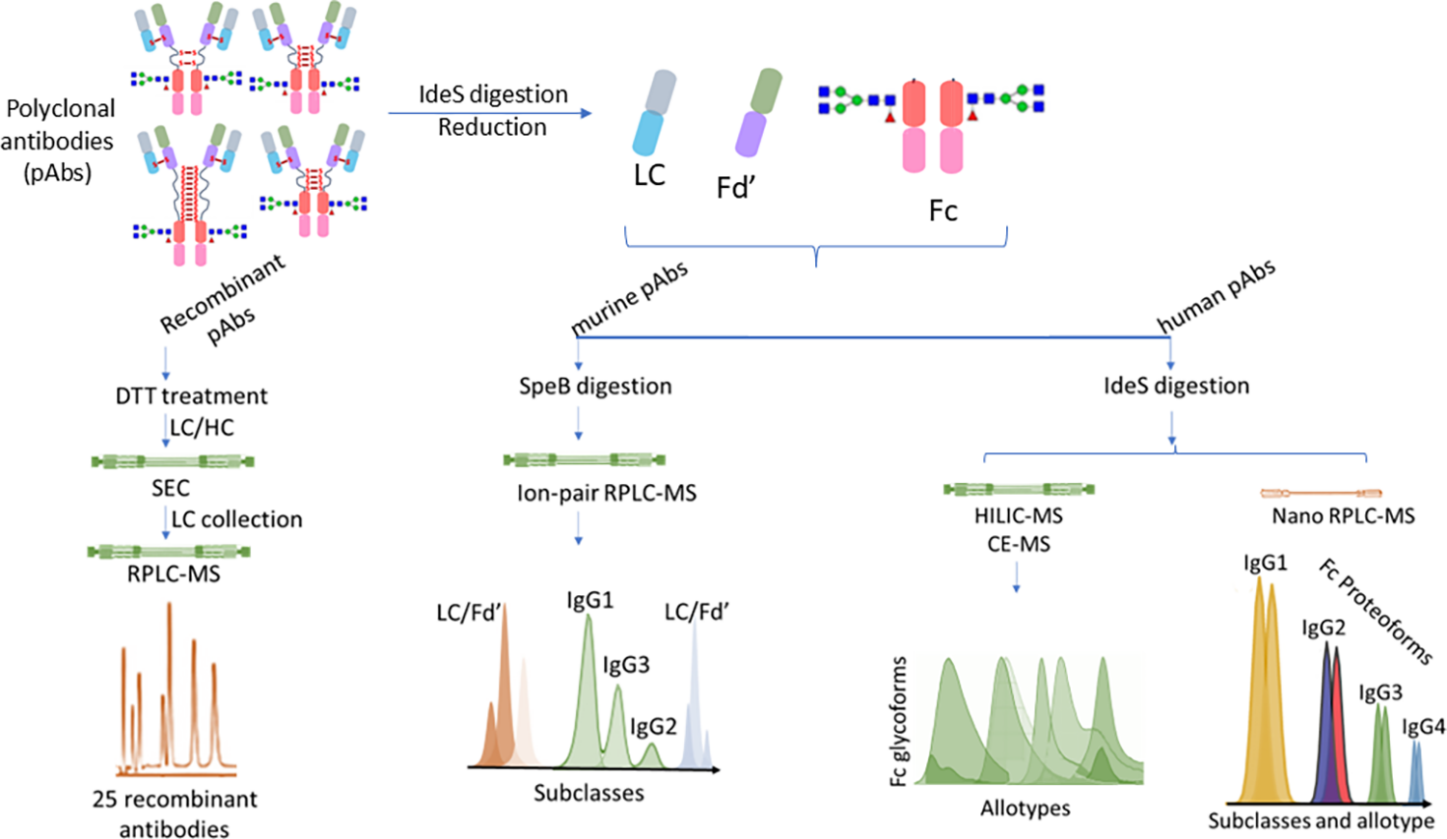
Schematic representation of different middle-up/subunit analysis strategies.

Furthermore, a complementary middle-up approach has been applied to the characterization of pAbs to differentiate κ and λ light chains. Antibodies contain one of two light-chain isotypes—kappa (κ) or lambda (λ)—with each immunoglobulin incorporating exclusively one type. Barnidge et al. demonstrated a strategy in which pAb samples were reduced with DTT to separate heavy and light chains, after which the light chains were analyzed by micro-LC–ESI–Q-TOF MS to distinguish κ and λ populations. This approach enables rapid assessment of light-chain distributions and can contribute to improved understanding of immune deficiencies, autoimmune diseases, and antibody responses.^63^

### 4.4 Physico-Chemical Properties Analysis

A comprehensive physicochemical characterization of pAbs provides critical insights into their light and heavy chain composition, PTMs, free sulfhydryl groups, and disulfide bridges. To assess these physicochemical properties, a peptide-centric strategy—commonly known as the bottom-up approach—is most frequently employed. In this method, antibodies are enzymatically digested using one or more proteases (e.g., trypsin, chymotrypsin, Glu-C) to generate smaller peptide fragments suitable for detailed analysis. Zheng et al. performed a series of bottom-up proteomic analyses on crude pAbs isolated from mouse serum to investigate PTMs and sequence variations contributing to the microheterogeneity of the pAb samples.^55^ Furthermore, Bondt et al. investigated the IgG1 repertoire in serum samples from two healthy donors and eight critically ill patients using bottom-up de novo sequencing. The study aimed to elucidate the dominance of specific IgG1 clones, assess sequence variability, and identify unique sequences among the IgG1 clones.^64^ However, bottom-up antibody characterization inherently requires exceptionally high sequence coverage to achieve such detailed insights. Ideally, each amino acid residue should be represented by multiple overlapping and unique peptides to ensure accurate de novo sequencing, confident identification of sequence variants, and precise localization of PTMs.^65^ In addition to characterization of PTMs and disulfide bond, another important physicochemical property of pAbs is the carbohydrate content (glycan), including neutral sugars, amino sugars, and sialic acids. In fact, glycosylation of the Fc region of IgG significantly influences its effector functions, including the ADCC and the complement-dependent cytotoxicity (CDC). Detailed glycan profiling, with particular focus on the extent of mannosylation, galactosylation, fucosylation, and sialylation, is essential to determine the relative distribution of the predominant glycan species, typically G0, G1, G2. Moreover, the light chain is generally nonglycosylated; potential additional glycosylation site(s) may occur on the heavy chain and should be identified and characterized. The bottom-up approach has been widely employed as an analytical strategy to elucidate glycosylation. Nathaniel Washburn et al. identified undesired glycan modifications in three approved intravenous immunoglobulin (IVIg) products—Gammagard, Privigen, and Octagam—which were attributed to their respective formulations using glycopeptide mapping.^66^ Zheng et al. employed a bottom-up proteomic approach and identified distinct glycoform profiles among the pAb samples at the tryptic glycopeptide level. Among these, the Asn297 residue within the peptide EDYNSTLR was determined to be the most extensively glycosylated site across all three pAb samples.^55^ Interestingly, Wang et al. applied a high resolution nanoHILIC combined with HCD based MS/MS approach to resolve glycopeptides and differentiate IgG subclasses in plasma samples from ten healthy donors. Their analysis revealed strong glycopeptide signals for IgG1 and IgG2, while IgG3 and IgG4 exhibited comparatively lower abundances. Further examination of the subclass-specific glycan compositions uncovered subtle but notable differences. For example, sialylation levels were higher in IgG2 (21%), IgG3 (30%), and IgG4 (26%) compared to IgG1 (14%), highlighting distinct glycosylation patterns among the IgG subclasses.^67^ A microflow LC platform coupled with a multinozzle electrospray ionization-parallel reaction monitoring (MnESI-PRM) workflow for subclass-specific monitoring of IgG N-glycopeptides has been reported. This approach enables quantitative analysis of Fc glycopeptides by integrating transitions derived from both glycosidic and peptide bond fragmentation, allowing precise monitoring of glycoforms. Using this method, up to 13 representative glycoforms can be effectively tracked for each IgG subclass, including IgG3 and IgG4, providing a robust tool for detailed subclass-specific glycosylation profiling.^68^ Moreover, van Tol et al. demonstrated a glycopeptide-centered LC-MS method capable of quantifying both N- and O-glycosylation in IgG isotypes and subclasses. LC separation of the different peptide clusters avoided overlapping masses. A nanoESI interface enhanced with dopant-enriched nitrogen gas confers supreme sensitivity and sufficient glycopeptide selectivity to obtain a deep coverage of microheterogeneity, This approach was applied to IgGs isolated from 160 clinical samples, including women from the Pregnancy-induced Amelioration of Rheumatoid Arthritis (PARA) cohort as well as healthy controls, enabling detailed characterization of glycosylation patterns across a large and clinically relevant population.^69^ In addition to the LC-MS approach, CE-MS is a well-established method for analyzing glycans and glycopeptides in IgG samples. Kammeijer et al. utilized CE-MS for glycopeptide analysis to distinguish and separate α2,3- and α2,6-sialylated glycans in recombinant IgG and IgG samples isolated from human serum. Their analysis revealed a clear distinction in sialylation patterns: α2,3-sialylated glycopeptides were detected exclusively in the recombinant IgG sample, whereas α2,6-sialylated glycopeptides were predominantly present in IgGs from human plasma. This finding reflects the differences in glycosylation machinery between recombinant expression systems and human cells, where recombinant systems often produce nonhuman or less common sialylation linkages, while human plasma IgGs predominantly carry the naturally occurring α2,6-sialylation.^70^ Furthermore, the CE-MS technique has also been widely applied for both qualitative and quantitative profiling of N-glycans in IgGs. For instance, Marie et al. developed a high-sensitivity, label-free workflow using CZE-MS to profile native, underivatized N-glycans released from five different types of purified human serum IgG. The method demonstrated high sensitivity and resolution, making it a powerful tool for detailed glycomic analysis of IgG samples.^71^ A comprehensive glycan analysis of IgGs using multiple analytical approaches, including CE, MALDI-TOF MS, and LC-MS/MS, at both the glycopeptide and released-glycan levels has been carried out. The study demonstrated that nanoLC-MS/MS with stepped-energy higher-energy collisional dissociation (HCD) offers the resolution and sensitivity required to accurately characterize site-specific glycoform distributions across individual IgG subclasses from human serum.^72^ Furthermore, Rosina et al. applied a bottom-up mass spectrometry approach to investigate O-glycosylation in polyclonal IgG3 from six human serum samples. The study combined nanoLC–ESI–ion trap MS/MS with electron-transfer dissociation (ETD) and capillary electrophoresis–MS/MS (CE-MS/MS) using collision-induced dissociation (CID) to achieve site-specific characterization of O-glycans. This integrated strategy enabled precise localization of IgG3 O-glycosylation sites and demonstrated the effectiveness of bottom-up MS workflows for resolving glycosylation heterogeneity in complex polyclonal antibody samples.^73^

### 4.5 Assessment of Polyclonality and Immunochemical Properties

Assessment of the polyclonality of pAbs is a critical aspect in their therapeutic development. Previous studies have demonstrated that antibodies can undergo mild chemical modifications under specific protein-modifying conditions, such as exposure to ferrous ions, heme, or low pH. Structural investigations, including crystallographic analyses, have shown that antibodies can adopt alternative binding-site conformations, enabling interaction with unrelated antigens and effectively expanding the functional antibody repertoire.^74^ Moreover, immunoglobulin pretreatments applied during manufacturing—such as fractionation or virus-inactivation steps—may differentially impact subsets of clones, leading to batch-dependent functional outcomes. For example, distinct biological responses, including variations in neutrophil activation or cell death, have been reported following ferrous ion exposure of different immunoglobulin preparations.^75^ To evaluate polyclonality, the bottom-up proteomic approach employing hydrogen–deuterium exchange mass spectrometry (HDX-MS) has become one of the preferred techniques for mapping antibody epitopes on their corresponding antigens.^76^ HDX-MS measures the rate at which backbone amide hydrogens within a protein exchange with deuterium during the exposure of D_2_O. Because the exchange rate depends on factors such as hydrogen bonding and solvent accessibility, it reflects the protein’s conformational dynamics and structural flexibility.^77^ Zhang et al. employed an HDX-MS–based approach to identify epitopes on the Fab regions of pAbs interacting with the cashew nut allergen. The pAbs were purified from the serum of goats immunized with cashew nut extract, and monovalent Fab fragments were generated via papain digestion to minimize potential complications arising from large immune complex formation. Using this strategy, four distinct epitope regions were identified for the pAb fragments.^78^ Ständer et al. described an HDX-MS workflow for comprehensive epitope mapping using full-length IgGs. The approach was initially demonstrated on pAbs isolated from rabbits immunized with factor H-binding protein (fHbp) antigen of *Neisseria meningitidis*, enabling the identification of multiple epitopes on recombinant fHbp and providing detailed insights into the binding profiles and relative abundance of distinct antibody populations elicited by vaccination. This workflow not only allows precise epitope localization but also facilitates characterization of the heterogeneity within the pAb response.^79^ Furthermore, the method was extended to human pAbs generated in response to immunization with fHbp antigen, demonstrating the versatility of HDX-MS in mapping human pAb epitopes and providing a deeper understanding of the diversity and functional relevance of antibody populations elicited by vaccination.^80^ In addition to HDX-MS, surface plasmon resonance (SPR) has been employed to investigate antigen–pAb interactions. Yang et al. demonstrated a proof-of-concept study combining SPR and HDX LC-MS to detect antigen-specific antibodies in serum, using samples from immunized mice. Nine serum samples, collected from various mouse strains immunized with human IL-13, were analyzed to evaluate the performance of the combined approach. The integration of SPR and HDX LC-MS allows a comprehensive assessment of antibodies in serum, providing both quantitative and qualitative information. SPR offers measurements of antibody affinity and antigen-specific antibody levels, while HDX-MS delivers detailed insights into the structural interactions between serum antibodies and their corresponding antigens.^81^ In addition to HDX-MS and SPR, cryo-electron microscopy (cryo-EM) has emerged as a powerful structural analytical technique for characterizing antigen–antibody interactions, particularly in the context of pAbs interaction with virus particles.^82,83^ Cryo-EM provides high-resolution visualization of antibody–antigen complexes, allowing detailed analysis of overall architecture, conformational heterogeneity, and stoichiometry. For instance, Antanasijevic et al. employed a cryo-EM approach for rapid structural analysis of pAb responses elicited by enteroviruses using intact coxsackievirus A21 (CV-A21) that are readily targeted by antibodies.^84^

### 4.6 Purity, Impurities, and Contaminants

Due to their production from large pools of human or animal plasma, pAbs products often contain a heterogeneous mixture of plasma proteins (apolipoprotein H, transferrin, albumin), degradation products, and potential infectious agents, even after extensive purification and viral inactivation steps.^85^ Assessing these contaminants and impurities is a critical aspect of quality control in pAb manufacturing. To ensure product safety and consistency, several advanced analytical approaches have been developed and implemented for the detection, characterization, and control of these potential contaminants. Gerber et al. developed and implemented an immunoaffinity chromatography (IAC) method to remove isoagglutinins from a chromatographically purified intravenous immunoglobulin (IVIg) product at an industrial scale.^86^ In addition to IAC, the BU proteomic approach is one of the preferred analytical methods for assessing impurities or contaminants in pAb products. Lackner et al. utilized a BU proteomic workflow coupled with MALDI-TOF mass spectrometry to detect impurities such as apolipoproteins in IgG products.^87^ In another study, Branovic et al. demonstrated that IEX chromatography, particularly anion-exchange chromatography, can be effectively integrated with protein G affinity purification to enhance the removal of host protein contaminants. Using monolithic disk columns, their two-step chromatographic process enabled efficient capture and elimination of residual plasma proteins, including transferrin and albumin, from IgG preparations.^88^ In addition to protein and viral impurities, process-related impurities such as caprylic (octanoic) acid (CA) are of significant concern during the manufacture of immunoglobulin products. CA is frequently employed as a fractionation agent for the purification of IgGs from both human and animal plasma; however, its complete removal from the final formulation must be ensured, and its absence must be reliably verified. To address this, Štimac et al. developed and validated a simple, sensitive, and rapid RP-LC method for the quantitative determination of CA in IVIg products, in accordance with good laboratory practice (GLP) principles.^89^

## 5 Conclusions

The fundamental requirement for any biotherapeutic product is the thorough monitoring and control of its CQAs, which directly determines product efficacy, safety, and consistency. Compared to mAbs, therapeutic pAb products derived from large pools of animal or human serum pose additional analytical challenges due to their inherent heterogeneity. pAbs comprise a diverse mixture of antibody populations that differ in subclass, isotype, and allotype, as well as in amino acid sequence, glycosylation patterns, and other PTMs. This molecular diversity significantly increases the complexity of their analytical characterization, making comprehensive structural and subclass distribution particularly demanding.

Advanced technologies in chromatographic materials and columns (i.e., wide pore size, new chemistries, core–shell technology), paralleled by instrumentation evolution (i.e., bioinert chromatographic systems and columns, bidimensional LC applications, advanced MS instrumentation and MS modes) have enabled their successful application in mAbs CQAs characterization. Inspired by mAbs analytical evolution, researchers can now take advantage of consolidated analytical approaches performed at the intact, subunit and peptide level for comprehensive evaluation of pAbs CQAs. A schematic representation of the increase and the number of publications found in literature over the past decade on the development of analytical methods of the main CQAs for pAbs characterization is given in Figure 4. pAbs structural complexity implies that none of these approaches or a single analytical technique is sufficient to characterize such highly heterogeneous products. For instance, intact mass or native MS analyses, valuable for providing an overview of molecular heterogeneity under near-native conditions for mAbs, often yield complex and overlapping spectra due to the coexistence of multiple antibody subclasses and isoforms within polyclonal preparations. These overlaps can obscure signals from individual species, making it difficult to accurately determine subclass distribution, sequence variants, or proteoform-specific attributes. Middle-up approaches are currently the most promising single-workflow strategy for subclass differentiation and relative quantification of subpopulations: they simplify the mass/charge space enough to separate and identify subclass-specific features (including major glycoforms and allotypic variants) while retaining more structural context than bottom-up methods. However, middle-up methods remain insufficient to fully describe the native architecture of pAbs i.e., the exact pairing combinations of heavy and light chains present in intact immunoglobulin molecules.

**Figure 4:**
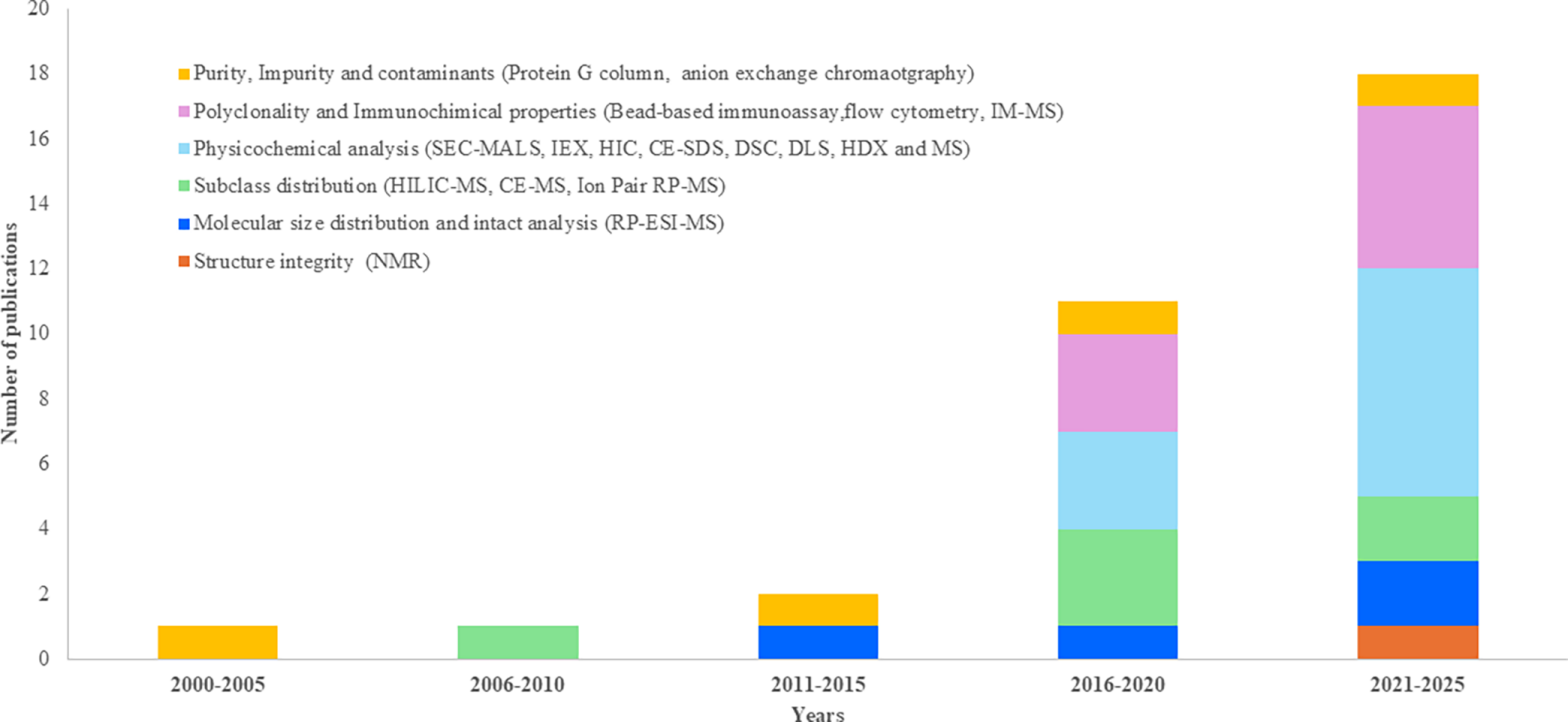
Trend of scientific publications on pAbs analytical characterization discussed in this study, from 2000 to 2025. Papers are grouped into 5-years windows mentioning the specific CQA profiled in each study.

In our opinion, new analytical strategies such as combinations of complementary and orthogonal analytical platforms (nanoLC and CE coupled with IM-MS) are essential to obtain a complete understanding of their molecular structure such as isotypes, subclasses, and glycosylation profile as well as product quality. One opportunity that remains largely unexplored for pAbs is the application of multidimensional chromatography, an approach that has been successfully implemented for the in-depth characterization of mAbs. In the case of mAbs, multidimensional chromatographic workflows integrate complementary separation techniques—such as SEC, CEX, hydrophobic interaction chromatography (HIC), and RP-LC coupled to MS. These strategies enable the combination of intact, middle-up and bottom-up analyses to comprehensively assess critical quality attributes, including size, charge, and hydrophobic variants.^90^

A key future direction in pAb product development is the increasing adoption of analytical procedures and tools capable of addressing different classes and subclasses of biomolecules, moving toward horizontal analytical approaches instead of multiple product-specific methods for similar biopharmaceutical products. The ICH has advanced a global regulatory initiative to harmonize reagents, analytical methodologies, and pharmaceutical development strategies, placing strong emphasis on Quality by Design (QbD). The core principles underpinning QbD are articulated in the ICH guidelines Q8–Q11 ^91^ which collectively encompass pharmaceutical development, quality risk management, pharmaceutical quality systems, and lifecycle management. Several biopharmaceutical companies, including Roche/Genentech, have successfully licensed therapeutic recombinant mAb products developed using QbD principles.^92^

Despite the use of risk management approaches such as AQbD is still in its infancy,^93^ this approach might be beneficial to the analysis of more complex IGg samples as therapeutic pAbs. The application of QbD—supported by state-of-the-art analytical methodologies, risk-based control strategies, and enhanced process understanding—could provide a rational pathway to systematically address pAb product variability. Consequently, adopting AQbD principles for pAb development could facilitate regulatory acceptance, ensure consistent clinical performance, and accelerate the introduction of pAbs as reliable and well-controlled biotherapeutic products.

Another powerful perspective can be seen looking at the recent advances in high-resolution mass spectrometry, particularly IM-MS, an additional gas-phase separation dimension based on molecular size, shape, and charge can be achieved. Furthermore, emerging MS-based techniques such as limited charge reduction offer additional opportunities to simplify complex spectra and enhance the structural characterization of pAb products. Collectively, the integration of multidimensional chromatography with advanced MS technologies represents a powerful and promising analytical framework for the comprehensive characterization and control of pAbs.

In addition to advances in analytical workflows, there will be a growing need for specialized algorithms to process data and for chemometric tools to analyze it. Recent advances in artificial intelligence (AI) and their application to data analysis and interpretation offer significant potential for the characterization of highly complex biological samples.^94^ In particular, AI-driven approaches can address challenges associated with the analysis of large, heterogeneous data sets generated by advanced MS workflows. For example, a recent study demonstrated the application of polySeq AI software to bottom-up proteomic analyses for de novo sequencing of antibody mixtures. Although this work represents a proof of concept, it highlights the transformative potential of AI-based tools to enable sequence-level characterization of pAb products.^95^ Analytical approaches in combination with AI tool will not only enhance our understanding of product heterogeneity but also facilitate regulatory compliance, ensuring consistent product quality and improved therapeutic outcomes. Looking ahead, we expect to see an increasing production of recombinant pAbs. Compared with conventional polyclonal preparations, recombinant pAbs may provide improved consistency, greater scalability, and tunable specificity, reflecting recent advances in therapeutic antibody development. Moreover, this more standardized production can facilitate the full characterization of pAbs.
